# Combined Effect of Extraction and Purification Conditions on Yield, Composition and Functional and Structural Properties of Lupin Proteins

**DOI:** 10.3390/foods11111646

**Published:** 2022-06-02

**Authors:** Sara Albe-Slabi, Odile Mesieres, Christelle Mathé, Mbalo Ndiaye, Olivier Galet, Romain Kapel

**Affiliations:** 1 Groupe Avril, 75008 Paris, France; sara.albe-slabi@groupeavril.com (S.A.-S.); mbalo.ndiaye@groupeavril.com (M.N.); olivier.galet@groupeavril.com (O.G.); 2 Laboratoire Réactions et Génie des Procédés, Université de Lorraine, CNRS, LRGP, 54000 Nancy, France; odile.mesieres@univ-lorraine.fr (O.M.); christelle.mathe@univ-lorraine.fr (C.M.)

**Keywords:** lupin protein, extraction, isoelectric precipitation, ultrafiltration, functional properties, structure

## Abstract

Lupin meal presents great potential as an alternative plant-based source of proteins for human nutrition. In the present work, different conditions of extraction and purification were evaluated for production of lupin protein isolates. The results showed that the protein extraction yield was comparable at acidic and conventionally used alkaline extraction pH (37% vs. 40–45%, respectively). Proteins extracted were principally composed of globulins. The ionic strength negatively impacted the protein extractability at pH 2, whereas no significant differences were observed between extractions at 20 to 50 °C. The selected extraction conditions (pH 2 and 7) combined with purification by isoelectric precipitation or ultrafiltration process generated the isolate-grade products. Interestingly, further characterization revealed a partial denaturation of proteins extracted at pH 2 resulting in loss of protein solubility at pH 6 and 7 (10–50%), modifications in secondary structure, lower thermal stability, and formation of protein aggregates. However, foaming and emulsifying properties were generally similar for almost all lupin isolates. Further investigation might be of interest with regard to the extraction behaviours and structural and functional properties of specific lupin protein fractions.

## 1. Introduction

The growing global population combined with socio-economic changes is leading to an increase in the demand for plant proteins for human nutrition. In recent years, much research has been aimed at developing new, high quality, and functional plant-based protein food products [1,2,3]. Nowadays, plant protein sources used in food production in France are generally limited to wheat, soybean, and pea [4]. However, it is necessary to exploit alternative plant-based protein sources.

White lupin (*Lupinus albus* L.) is legume crop widely cultivated in Australia, Russia, and Poland with global production yield of over 1 million ton in 2019 [5]. Its seeds are particularly rich in dietary proteins (35–50% on a dry matter basis as reported by others [6,7,8]). Like other legumes, the majority of lupin storage proteins are composed of globulins (about 90% of total storage proteins) [9]. Four main globulin fractions can be distinguished: “legumin-like” α-conglutin (11S, 330–440 kDa), “vicilin-like” β-conglutin (7S, 143–260 kDa), δ-conglutin (2S, 13 kDa), and ɤ-conglutin (7S, 200 kDa) [6,10,11,12]. With the exception of sulphur-containing amino acids, lupin proteins fulfil the FAO requirements [13] concerning amino acid composition for human consumption. Furthermore, they are particularly rich in lysine in contrast to many other plant protein sources [14,15]. Lupin proteins are also known to have digestibility comparable to or better than casein and other legumes [14,15,16]. Furthermore, previous studies have pointed out interesting functional properties such as solubility, foaming, and emulsifying properties and water- and oil-binding capacities [15,17]. Therefore, lupin proteins have great potential to be used as protein isolates for various applications in the food industry [18].

The process of production of plant-based protein isolates is composed of solid/liquid extraction followed by protein purification. The aim of this process is to produce a protein-rich product (>90% purity on a dry matter basis) with low content of non-protein and antinutritional compounds (fibres, alkaloids, lectins, phenolic compounds, phytate, etc.) [2,19].

At the present time, three principal strategies are usually investigated for production of protein isolate from lupin seeds and meal. Due to its simplicity and possible applicability in the food industry, the most commonly implemented process is alkaline extraction at pH 8–9 and further protein purification by isoelectric precipitation at pH 4.5–5 [7,10,11,14,15,20,21,22]. The global yield of production of lupin proteins achieved with this method ranged from 20 to 50% [7,14]. However, as demonstrated by Muranyi and co-workers [21,23], this strategy leads to denaturation of the protein native structure that may limit protein functionality. Therefore, some authors have recently explored the performances of alternative methods for protein purification, such as the process membrane method. Indeed, compared to isoelectric precipitation, ultrafiltration is an efficient process to produce lupin protein concentrates with higher protein recovery (90% vs. 50% for ultrafiltration and isoelectric precipitation, respectively) and remarkably improved solubility [8,14]. The final strategy employed for production of lupin protein isolate is salt-induced extraction followed by diluted precipitation, also called micellization [11,15,21,23]. Although, this method was found to prevent unfolding and irreversible modifications in protein structure, it requires a huge amount of salt, which may be an important hindrance to the industrialization of the process [21,23].

Interestingly, the recent study by Fontanari et al. [20] demonstrated the high extractability of lupin proteins in acidic medium (60–65% at pH 1–2) comparable to those obtained with the conventionally used alkaline extraction. However, there are limited data currently available in the literature concerning the impact of process conditions on the yield and quality of lupin proteins. Hence, the aim of this work was to study the effect of several extraction parameters (pH, NaCl concentration and temperature) on lupin protein extractability and composition. In the second part of the study, the process yield and the quality of lupin proteins purified by two different purification methods, ultrafiltration and isoelectric precipitation, were compared.

## 2. Materials and Methods

### 2.1. Materials and Reagents

Lupin meal from white lupin seed (*Lupinus albus* L.) was supplied by Olead (Pessac, France). The initial protein and fat content were 47.4 and 0.4% on a dry matter basis, respectively. The sodium chloride (NaCl, CAS 7647-14-201), sodium hydroxide (NaOH, CAS 1310-73-2), sodium citrate dihydrate (C_6_H_5_Na_3_O_7_·2H_2_0, CAS 6132-04-3) and citric acid (C_6_H_8_O_7_·H_2_O, CAS 5949-29-1) were from VWR (Darmstadt, Germany). The hydrochloric acid (HCl, CAS 7647-01-0) was purchased from Carlo Erba (Milan, Italy). Boric acid (H_3_BO_3_, CAS 10043-35-3), sodium tetraborate decahydrate (B_4_Na_2_O_7_·10H_2_O, CAS 1303-96-4), sodium phosphate monobasic monohydrate (NaH_2_PO_4_·H_2_O, CAS 10049-21-5) and sodium phosphate dibasic dodecahydrate (Na_2_HPO_4_·12H_2_O, CAS 10039-32-4) were from Merck KGaA (Darmstadt, Germany).

### 2.2. Effect of Extraction Conditions

Solid/liquid extraction of lupin proteins was performed according to the protocol determined by Sussmann et al. [24] with some modifications. In brief, lupin meal was mixed with distilled water or an aliquot of NaCl solution (0.25 or 0.5 mol·L^−1^), respecting the solid/liquid ratio of 1:10 (*p*/*v*). The temperature was set at 20 or 50 °C. The pH was adjusted to a given value (2–10 ± 0.05) using 1 mol L^−1^ NaOH or HCl solution. The suspension was stirred at 300 rpm for 60 min and, if necessary, the pH was adjusted again. Then, suspension was centrifuged at 15,000× *g* at 20 °C for 30 min. The obtained supernatant was additionally filtered using Whatman paper (17–30 µm of pore size, 190 × 0.17 mm). The obtained liquid phase was referred to as the liquid extract.

### 2.3. Preparation of Lupin Protein Isolates

**Figure 1:**
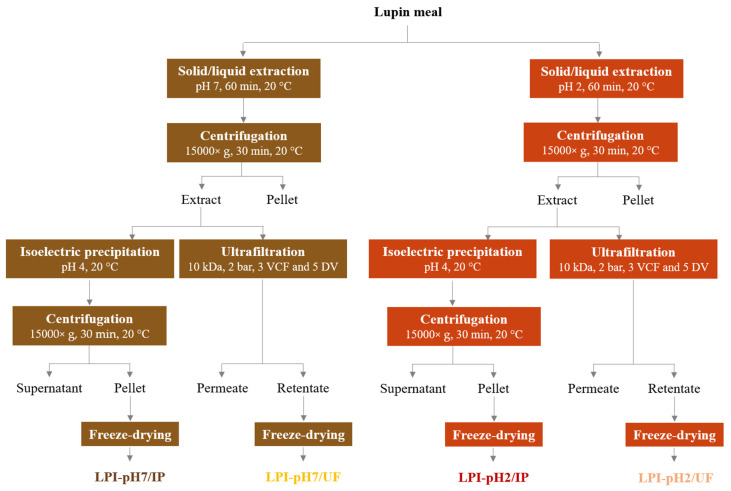
Schematic process of production of lupin protein isolates.

Four lupin protein isolates were prepared according to the process depicted in the scheme in Figure 1. The first step, based on solid/liquid extraction at pH 2 or pH 7, was common for the production processes of all isolates. The detailed protocol for this step was described in Section 2.2.

#### 2.3.1. Purification by Isoelectric Precipitation

The 1500 mL of lupin proteins extracted at pH 2 or 7 was treated with isoelectric precipitation. To maximize the purification yield, the pH of the extract was adjusted to pH 4 (±0.05) using a solution of 1 mol·L^−1^ NaOH or HCl. The suspension was stirred at 300 rpm for 20 min at room temperature and then centrifuged at 15,000× *g* for 30 min at 20 °C. After separation, the precipitate was washed two times with approximately 350 mL of ultrapure water and centrifuged again. The pellet was then freeze-dried. The process yielded lupin protein isolates referred to as LPI-pH2/IP or LPI-pH7/IP, respectively, in terms of the pH of extraction.

#### 2.3.2. Purification by Ultrafiltration

Ultrafiltration of the liquid extract was performed using an Akta Flux^®^ 6 system coupled with 10 kDa hollow fibre cartridge (200 cm^2^), all supplied by GE Healthcare (Chicago, IL, USA). As was confirmed in the trial test (Table 1), this membrane cut-off yielded a high retention rate of lupin proteins (1.0) and suitable flux (0.09 mL/min/cm^2^). The 1500 mL of aqueous extract was first concentrated by 3 volumetric concentration factors (VCF) to reach 500 mL of retentate. Then, the retentate was washed with 5 diafiltration volumes (DV) using ultrapure water. Transmembrane pressure was maintained at 2 bars during all processes. The retentate was collected and freeze-dried. Depending on the extraction pH, the obtained isolates were named LPI-pH2/UF or LPI-pH7/UF, respectively.

### 2.4. Analytical Methods

#### 2.4.1. Determination of Protein Content and Protein Process Yields

The total nitrogen content in solid and liquid samples was determined with the Kjeldahl method following the procedures of AOAC method 991.20 [25]. In brief, 4 mL of 96% H_2_SO_4_ (*v*/*v*) and approximately 10 mg of catalyst was added to 0.5 mL or 30 mg of sample and the mixture was mineralized at 450 °C for 150 min. Then, the digestate was distilled with 32% NaOH (*w*/*v*) and treated with 3% boric acid (*w*/*v*) solution. Finally, the mixture was titrated against 0.01 mol·L^−1^ HCl. A blank consisted of a non-protein sample. A nitrogen–protein conversion factor of N × 5.7 was used, as previously reported for lupin proteins [7,23,24,26]. Protein extraction yield and purification yield were calculated as the percentage of the weight of the crude proteins in the extract or in the final product to the weight of proteins in the raw material or in the extract, respectively. Protein recovery was calculated as the ratio between the protein weight in the final products and the extract.

#### 2.4.2. Electrophoresis on Gel

Sodium dodecyl sulphate–polyacrylamide gel electrophoresis (SDS-PAGE) under reducing conditions was performed according to the Laemmli method [27]. To do so, the aqueous extract was diluted, mixed with Laemmli buffer containing 2% β-mercaptoethanol (*v*/*v*) and then heated at 95 °C for 5 min. Proteins were separated on gel composed of 5% stacking and 17% resolving gel, applying an electric current of 20 mA per gel. Polypeptide SDS-PAGE Standard (26.6–6.5 kDa) from Bio-Rad (Hercules, CA, USA) was used as a molecular weight marker. After migration, gels were stained with Coomassie Brilliant Blue and destained overnight in 10% acetic acid solution (*v*/*v*).

### 2.5. Characterization of Isolated Proteins

#### 2.5.1. Protein Purity and Solubility

To measure the protein purity in the lyophilized powder [28], a solution with a concentration of 5 g·L^−1^ of isolated protein powder in 0.1 mol·L^−1^ NaOH was prepared and then centrifuged (1100× *g*, 10 min, 20 °C). The concentration of proteins in the supernatant was determined with the Kjeldahl method as described in Section 2.4.1. The results were expressed as the percentage of protein content in the lyophilized powder on a dry matter basis.

For protein solubility, a stock solution of proteins in distilled water was prepared at a final concentration of 5 g·L^−1^. The pH of the solution was adjusted to 3, 5, 7 and 9 using 0.1 mol·L^−1^ HCl or NaOH solution. The added volume was noted and considered in calculations. The given pH was kept at a constant value (±0.05) for 10 min under agitation at about 300 rpm. Then, the suspension was centrifuged at 15,000× *g* at 20 °C during 30 min. The protein content in the supernatant was assessed using the Kjeldahl method as described in Section 2.4.1. The solubility of lupin proteins as a function of pH was calculated as the percentage of the protein weight in the supernatant compared to the protein weight in initial solution.

#### 2.5.2. Measurement of Colour

The colour of the lupin protein isolates was determined in aqueous solution as previously described by Albe-Slabi et al. [28]. For this purpose, samples were prepared at a concentration of 1% (*w*/*w*) of proteins in 0.5 mol·L^−1^ of phosphate buffer, pH 7.0. The colour was measured using Lovibond PFX195 (Tintometer, Amesbury, UK) and considering the CIE L*a*b* uniform space scale, where the term L was the index of lightness, a* was a colour coordinate from green to red and b* was a colour coordinate from blue to yellow.

#### 2.5.3. Functional Properties

Foaming and emulsifying properties of isolated lupin proteins were evaluated according to the method proposed by Vioque et al. [29] with modifications.

For foaming stability and capacity, a protein solution at a final concentration of 1% (*w*/*v*) was prepared in 0.5 mol·L^−1^, pH 7 sodium phosphate buffer. The volume of 20 mL was then mixed at 10,000 rpm at room temperature for 5 min using the Ultra-Turrax^®^ T25 digital homogenizer from IKA (Staufen im Breisgau, Germany). Foaming capacity (*FC*) was calculated according to Equation (1):

(1) $$FC=\frac{V_{m}}{V_{i}}\times 100$$

where *FC* is the foaming capacity, *V_m_* is the obtained foam volume after mixing and *V_i_* is the initial protein solution before mixing. Foaming stability was determined by measuring the percentage of foam volume left after 5, 15, 30, 60 and 120 min.

Emulsifying properties were measured using 5 mL of protein solution in 0.5 mol·L^−1^, pH 7, sodium phosphate buffer at a concentration of 5% (*w*/*v*). The solution was mixed with 2.5 mL of sunflower oil at 10,000 rpm at room temperature for 30 s using the Ultra-Turrax^®^ homogenizer. Then, 2.5 mL of oil was added and mixed again for 90 s. After this, the mixture was centrifuged at 1100× *g* for 5 min at 20 °C. The emulsified layer was recorded and the emulsifying capacity (*EC*) was calculated as follows (Equation (2)):

(2) $$EC=\frac{V_{e}}{V_{i}}\times 100$$

where *EC* is the emulsifying capacity, *V_e_* is volume of the emulsified layer remaining after centrifugation and *V_i_* is the volume of the mixture before centrifugation. For measurement of emulsion stability, the mixture obtained after centrifugation was placed in a water bath heated at 85 °C for 15 min and then the mixture was centrifuged again using the same parameters. The stability of the protein emulsion was calculated as a ratio of the emulsified layer before and after heating. The results were expressed as a percentage.

#### 2.5.4. Structural Properties

- Circular Dichroism

The structural stability of lupin proteins against various pH values was analysed using a Chirascan Plus device from Applied Photophysics (Leatherhead, UK). Following the procedures of Albe-Slabi et al. [28], protein solutions at a final concentration of 1 g·L^−1^ of proteins at pH 3 (10 mmol·L^−1^ citrate buffer), pH 7 (10 mmol·L^−1^ sodium phosphate buffer) and pH 9 (10 mmol·L^−1^ borate buffer) were prepared. Prior to measurement, prepared solutions were filtrated (0.22 µm) and centrifuged at 36,000 rpm at room temperature for 10 min. During analysis, instruments were maintained under a constant flow of nitrogen gas. The temperature was set at 20 °C. The blank assay corresponded to the appropriate buffer solution. The far-UV spectra were recorded from 180 to 280 nm. All spectra were repeated at least in triplicate and a mean was calculated. Spectra were converted into mean residue ellipticity (Ɵ_MRE_) using protein concentration as determined by the Kjeldahl method (see Section 2.4.1).

- Differential Scanning Calorimetry

The evaluation of the thermal denaturation of lupin proteins was carried out through differential scanning calorimetry analysis following the method described by Albe-Slabi et al. [28]. For this purpose, a Microcal VP-DSC from Malvern Panalytical (Worcestershire, UK) was used. Prior to analysis, the protein solution at a concentration of 2 g·L^−1^ in 10 mmol·L^−1^ sodium phosphate buffer, pH 7, was filtered (0.22 µm) and degassed. A blank assay phosphate buffer was used. A thermogram was recorded during the linear heating (1 °C per min) from 20 to 130 °C. From the obtained data the temperature of denaturation (Tm) and the enthalpy calorimetry ∆Hcal (kcal/mol/°C protein) were calculated.

- Dynamic Light Scattering

Volumetric distribution of particle size of lupin proteins was assessed using a Zeta Sizer Nano-S purchased by Malvern Instruments (Worcestershire, UK). For this purpose, the procedures described previously by Albe-Slabi et al. [28] were used. In brief, protein solutions at a final concentration of 1 g·L^−1^ at pH 2 (10 mmol·L^−1^ hydrochloric acid-potassium chloride buffer), pH 7 (10 mmol·L^−1^ sodium phosphate buffer) and pH 9 (10 mmol·L^−1^ borate buffer) were used. Prior to measurement, the prepared solutions were filtrated (0.22 µm) and centrifuged at 36,000 rpm at room temperature for 10 min. During analysis, the temperature was set at 25 °C. For all analyses, between 13 and 17 scans were recorded and the mean distribution was determined.

### 2.6. Statistical Analysis

All measurements were performed at least in triplicate (*n* = 3) and data were reported as the mean with standard deviation (±). Analysis of variance using the F-test with a confidence level of 95% (*p*-value < 0.05) was applied to assess statistical differences between independent samples. Results of statistical analysis are presented with letters and samples with common letter are not significantly different.

## 3. Results and Discussion

### 3.1. Effect of Extraction Conditions on Extraction Yield and Protein Composition

#### 3.1.1. Effect of pH

**Figure 2:**
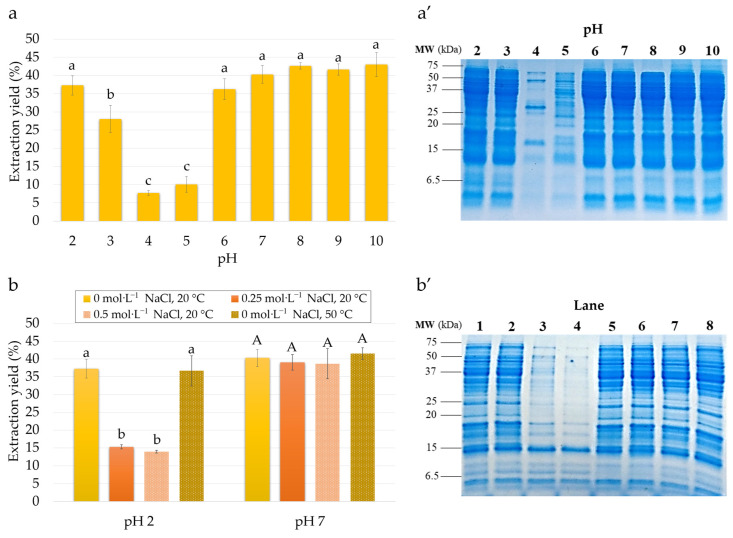
Effect of pH (2–10) on extraction yield of lupin proteins (**a**) and SDS-PAGE gel under reducing conditions for corresponding lupin protein extracts (**a′**). Effect of NaCl concentration (0, 0.25 and 0.5 mol·L^−1^) and temperature (20 and 50 °C) on extraction yield of lupin proteins at pH 2 and 7 (**b**). SDS-PAGE gel under reducing conditions for lupin protein extracts (**b′**) obtained at pH 2/0 mol·L^−1^ NaCl at 20 °C (lane 1), pH 2/0 mol·L^−1^ NaCl at 50 °C (lane 2), pH 2/0.25 mol·L^−1^ NaCl at 20 °C (lane 3), pH 2/0.5 mol·L^−1^ NaCl at 20 °C (lane 4), pH 7/0 mol·L^−1^ NaCl at 20 °C (lane 5), pH 7/0 mol·L^−1^ NaCl at 50 °C (lane 6), pH 7/0.25 mol·L^−1^ NaCl at 20 °C (lane 7) and pH 7/0.5 mol·L^−1^ NaCl at 20 °C (lane 8). Results of statistical analysis are presented with letters and samples with common letter are not significantly different.

Figure 2a presents the effect of extraction pH (2 to 10) on lupin protein extraction yield. According to these results, from pH 7 to 10 the extraction yield was high (about 41–43%) and did not differ significantly. The extractability of proteins decreased slightly at pH 7 (40.67 ± 2.31%) and 6 (36.00 ± 2.65%). The lowest extractability of lupin proteins was observed at pH 4 (7.67 ± 0.58%) and 5 (10.33 ± 2.31%). Surprisingly, under strong acidic conditions the extraction yield of proteins rose again to 28.00 ± 3.61% at pH 3 and 37.33 ± 2.52% at pH 2. The present results are in line with prior work showing high extraction of lupin proteins at pH 8–9 [7,14,15,20]. Poor extractability of lupin proteins between pH 4 and 5 (about 5–15%) has also been reported by other authors [15,20]. Indeed, the minimum lupin extractability covers the isoelectric point (iP) of most lupin conglutin, determined to be between pH 4.3 and 6.2 [6,10,12]. Overall, this extraction profile coincides well with the general solubility curves of protein from other plant-based sources at mild acidic, neutral and alkaline pH [30]. However, the high extraction yields in a strong acidic medium that were observed for lupin proteins are rather exceptional as other plant-based sources show poor extraction yields at low pH [30,31,32]. 

To evaluate the impact of extraction pH on the presence of specific proteins in lupin extracts, the electrophoretic analysis was performed. Figure 2a′ presents the SDS-PAGE gels under reducing conditions for lupin proteins extracted at pH from 2 to 10. As can be seen, extraction at pH from 6 to 10 yielded the same protein pattern. The gel revealed many heterogeneous bands with apparent molecular weights from about 3 to 70 kDa. This electrophoretic profile fit well with the molecular weight pattern of lupin conglutins. The detected bands were attributed to the subunits and polypeptides chains of α- (19–46 kDa), β- (19–60 kDa), ϒ- (17 and 29 kDa) and δ-conglutin (4 and 9 kDa) [6,10,12]. Similar results from SDS-PAGE analysis were previously demonstrated by Wong et al. [16] for proteins extracted at pH 8.5 from Lupinus angustifolius. In contrast, the significant decrease in the intensity of α-, β- and δ-conglutin bands in the extracts at pH 4 and 5 was noted. It was particularly visible for pH 4 where only three bands at about 17, 30 and 50 kDa, identified as ϒ-conglutin, were primarily detected. This lupin globulin has a basic nature (iP at pH 7.9) and thus, in contrast to other globulins, is soluble in this pH range [12]. The predominant extraction of ϒ-conglutin at pH 4.5 has also been reported by Wong et al. [22]. In addition, the results obtained by Sironi et al. [33] suggested that, in contrast to other lupin globulins, ϒ-conglutin remained soluble in acidic medium after protein precipitation. Regarding the extracts at pH 2 and 3, the electrophoretic profile showing the presence of major conglutins was similar to those of the extracts at pH 6 to 10. This finding was astonishing considering the selective extraction of albumins in acidic pH previously reported for many other plant proteins [28,31,34]. Indeed, plant globulins generally have much lower structural resistance compared to albumins [35]. Consequently, acidic extraction below the iP of globulins leads to their denaturation, aggregation and entrapment in the plant matrix. The present results thus highlight the singular extraction behaviours of lupin globulins allowing their solubilisation from meal even below the iP.

Overall, the presented results showed that, as well as from the commonly used extraction at alkaline pH, strong acidic and neutral conditions also yield high extractability for lupin proteins. However, this extraction strategy has not yet been explored in the literature. Therefore, a more thorough analysis of proteins extracted at pH 2 and 7 and their characterization would be of great interest.

#### 3.1.2. Effect of NaCl Concentration

As was previously reported, ionic strength could be an extraction parameter considerable affecting the solubility of plant proteins from meal [30,31]. Therefore, the effect of NaCl concentrations ranging from 0 to 0.5 mol·L^−1^ on the extraction yield (Figure 2a) and composition (Figure 2b) of lupin proteins was also considered.

According to these results, no significant modification of lupin protein extractability at pH 7 was observed in the extraction without salt (40.67 ± 2.31%) compared to the extraction with salt addition (39.33 ± 2.08% and 38.33 ± 4.51% for 0.25 mol·L^−1^ and 0.5 mol·L^−1^ NaCl, respectively). These conditions yielded the same protein pattern (Figure 2b′) at all studied ionic strengths. In contrast, a negative impact of NaCl concentration on lupin protein extractability was noted at pH 2. Indeed, under these conditions the protein extraction yield decreased considerably, from 37.33 ± 2.52% without salt addition to 15.33 ± 0.58% at 0.25 mol·L^−1^ NaCl and 14.00 ± 0.00% at 0.5 mol·L^−1^ NaCl. As shown by the SDS-PAGE gel, the majority of lupin globulins were not extracted under higher ionic strengths (Figure 2b′). Only two bands were observed at about 10 and 5 kDa molecular weights, which were assigned to the heavy and light chains of δ-conglutin, respectively. Unlike other lupin globulins, this lupin globulin is a monomeric protein that probably has a more resistant structure against acidic conditions [12].

El-Adway et al. [15] have previously demonstrated the improvement in protein extraction yield from bitter and sweet lupin, from 20% at 0 mol·L^−1^ to 80% and 0.2 mol·L^−1^ NaCl. The authors attributed this observation to the “salting-in” effect. Similar conclusions have been drawn by Sussmann et al. [24], who reported an increase in the protein extractability from blue lupin (38% in 0.5 mol·L^−1^ NaCl vs. 23% in 0.2 mol·L^−1^ NaCl). However, this effect was primarily observed at pH 5, corresponding to the isoelectric region. Regarding oilseed proteins, in generally, addition of salt also improves the protein extraction yields at pH close to the neutrality. Indeed, in a recent study by Albe-Slabi et al. [30], the authors reported that the increase of ionic strength up to 0.5 mol·L^−1^ NaCl resulted in an increase of the extraction yield of globulins from 30% to 50% and albumins from 40% to 60%. Therefore, the results presented above emphasise the distinct extraction behaviour of lupin proteins, with no significant effect from NaCl addition at neutral pH on protein solubility and composition.

#### 3.1.3. Effect of Temperature

The effect of temperature (20 and 50 °C) on lupin protein extractability is depicted in Figure 2b. For both extractions, at pH 2 and 7, the recovery of proteins at 50 °C was high and comparable to that at 20 °C (about 37% for both temperatures at pH 2 and 42.5% vs. 40.5% for pH 7 at 50 and 20 °C, respectively). Therefore, the obtained results clearly demonstrated that the increase in temperature from 20 to 50 °C did not have a significant impact on the extractability of proteins from lupin meal under the studied conditions. A similar observation was drawn after the SDS-PAGE analysis (Figure 2b′), which showed that the heat treatment at pH 7 revealed no additional protein bands in the extractions at 20 and 50 °C. There was also no obvious difference in the SDS-PAGE gels at 20 and 50 °C for extraction at pH 2. Under all extraction conditions the signals of α-, β-, ϒ- and δ-conglutin were present and equally intense. The obtained results indicate clear support for the findings of Berghout et al. [36] showing a similar pattern in the SDS-PAGE gel for protein isolates extracted at pH 7 and 20 or 50 °C. In this study, the degradation of the signal of high-molecular-weight proteins occurred at the temperature closest to 90 °C. This weak effect was surprising, especially regarding other plant protein sources. Indeed, the increase in the temperature generally improves protein solubility from meal, as shown for rapeseed proteins by several authors [28,31,34].

Altogether, extraction at pH 2 and 7 yielded comparable protein recovery from lupin meal (about 40%). Under those conditions the maximal extraction yield of lupin proteins was achieved and the increase in NaCl concentration and temperature did not enable any improvement in their extractability. Hence, to reduce the cost of the production process and its harmful impact on the environment, the extraction of lupin proteins without addition of salt and at 20 °C should be privileged.

### 3.2. Combined Effect of Extraction pH and Purification Process on Protein Recovery, Composition and Color of Lupin Protein Isolates

**Figure 3:**
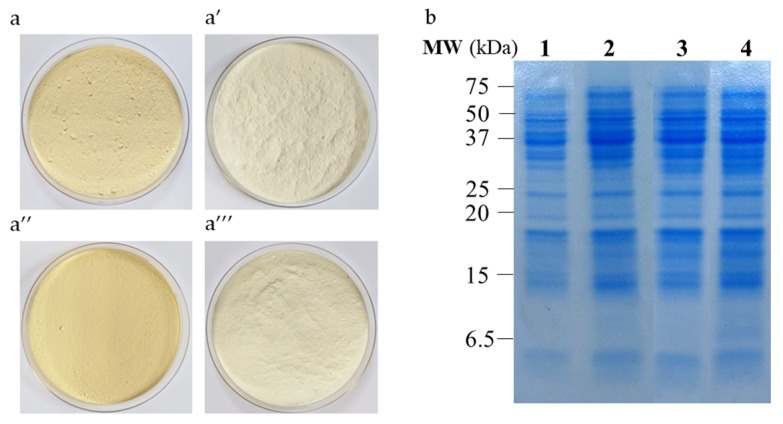
Colour of lupin protein isolates: LPI-pH7/IP (**a**), LPI-pH7/UF (**′**), LPI-pH2/IP (**a″**) and LPI-pH2/UF (**a‴**). SDS-PAGE under reducing conditions for lupin protein isolates (**b**): LPI-pH7/IP (lane 1), LPI-pH7/UF (lane 2), LPI-pH2/IP (lane 3) and LPI-pH2/UF (lane 4).

Table 2 shows protein yields and lupin protein composition after extraction (pH 2 or 7) and purification achieved by either isoelectric precipitation or ultrafiltration (LPI-pH7/IP, LPI-pH7/UF, LPI-pH2/IP and LPI-pH2/UF). According to these data, extraction yield of the scaled-up process was comparable for all conditions (37.1–40.4%). Regarding the purification yield, it ranged from 66.5 to 76.1% for both isoelectric precipitation and ultrafiltration. All applied processes yielded lupin proteins of isolate grade (95.7, 91.5, 91.2 and 100.3of purity on a dry matter basis for LPI-pH7/IP, LPI-pH7/UF, LPI-pH2/IP and LPI-pH2/UP, respectively). Their colour in solid state was light yellow (Figure 3a–a‴). In aqueous solution, isolates were characterized by an index of L* close to 100 (Table 3), indicating a high lightness. The a* and b* coordinates suggested that proteins produced by isoelectric precipitation had a slightly higher orange tone than those from ultrafiltration, which were more yellow. As shown by the SDS-PAGE in Figure 3b, these four isolates had also similar protein profiles corresponding to major lupin conglutins.

Surprisingly, Chew et al. [14] previously reported a higher purification yield for ultrafiltration using a 10 kDa membrane than for isoelectric precipitation at pH 4.5 (92% vs. 59%, respectively). However, the resulting protein purities were relatively low (75% and 67 % for ultrafiltrated and precipitated proteins, respectively). Usually, higher purification yields in ultrafiltration (70%) compared to isoelectric precipitation (about 30%) are observed for oleoproteaginous proteins that are richer in albumins. This protein fraction remains soluble in acidic medium, generating important losses during isoelectric precipitation. Albumins are thus more effectively recoverable from aqueous extract using the ultrafiltration process. Since lupin proteins are mainly composed of globulins, both purification methods should show similar performances.

### 3.3. Effect of Extraction Conditions on Yield and Protein Properties

#### 3.3.1. Solubility

**Figure 4:**
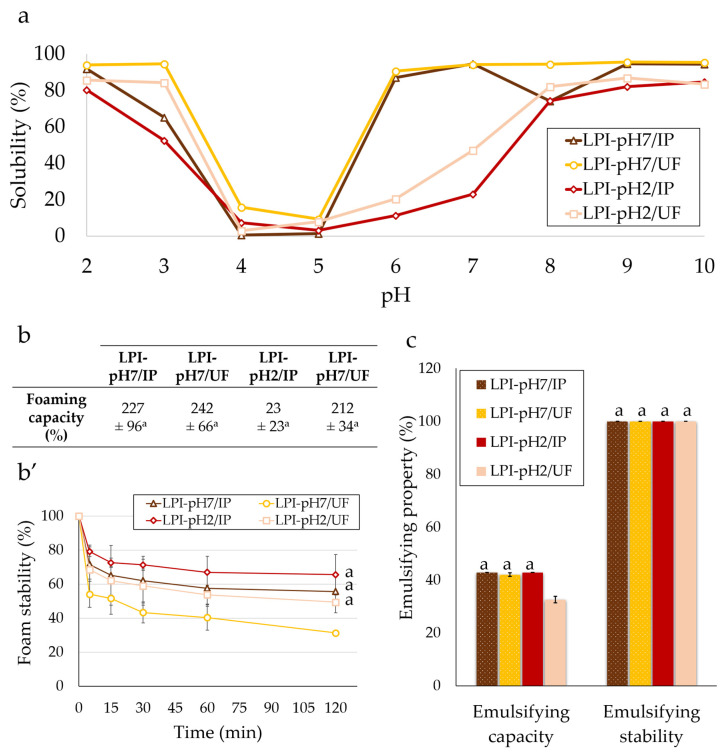
Solubility of lupin protein isolates as a function of pH from 2 to 10 (**a**) as prepared with: extraction at pH 7 and isoelectric precipitation (△); extraction at pH 7 and ultrafiltration (◯); extraction at pH 2 and isoelectric precipitation (◇); extraction at pH 2 and ultrafiltration (☐). Also shown is a comparison of the foaming capacity (**b**), foaming stability (**b′**), emulsifying capacity and stability (**c**) of lupin protein isolates at pH 7. Results of statistical analysis are presented with letters and samples with common letter are not significantly different.

The solubility of lupin protein isolates as a function of pH ranging from 2 to 10 is presented in Figure 4a. Generally, all lupin proteins were well-soluble at strong acidic (80–95%) and alkaline pH (95%). The lowest solubility for all lupin isolates was observed between pH 4 and 5 (0–15%). This solubility profile is characteristic of lupin proteins, as it has been previously described by other authors [8,14,37]. The U-shape curve is associated with the isoelectric point of lupin proteins, determined to be around pH 4–6 [6,10,12]. However, at mild acidic and neutral pH, a significant difference in the solubility of proteins depending on the extraction pH was noted. Indeed, the proteins extracted at pH 2 (LPI-pH2/IP and LPI-pH2/UF) were poorly soluble at pH 6 and 7 (10–50%), whereas the solubility of proteins obtained by extraction at pH 7 (LPI-pH7/IP and LPI-pH7/UF) reached about 90–95%. The observed variation can probably be explained by the partial denaturation of proteins exposed to the extreme acidic medium of the extraction process. Nonetheless, the solubility of lupin protein isolates presented in this work was considerably improved compared to those of Hojilla-Evangelista et al. [8] and Muranyi et al. [11,21], who reported about 25–50% solubility in lupin proteins at pH 6. Noteworthy, such a high solubility at mildly acidic pH is also exceptional in relation to other plant proteins. Indeed, as previously reported, proteins from other pulses, soybean and oilseeds usually have about 0–40% solubility around pH 6 [8,30,38].

#### 3.3.2. Foaming

Figure 4b,b′ show the foaming properties of lupin protein isolates. According to these results, all produced isolates exhibited almost identical foaming capacities (212–242%, Figure 4b). No significant difference was observed between them. Similarly, the stability of foam (Figure 4b′) was comparable across all isolates (50–65% of remaining foam volume after 120 min), except for LPI-pH7/UF, which produced the most unstable foam (about 35% over 120 min). In the study by Hojilla-Evangelista et al. [8], the forming properties of lupin protein concentrates prepared by alkaline extraction followed by isoelectric precipitation or ultrafiltration were also compared. In line with the present results, the authors pointed out the nearly identical foaming capacities of the tested concentrates. Also, the foam produced from concentrate obtained by extraction at pH 8 and ultrafiltration was less stable compared to precipitated proteins. On the other hand, close results for foaming capacity for acidic-soluble lupin proteins were also demonstrated for whatever purification method was used [22]. In a later study, Alu’datt et al. [39], investigating the functional properties of protein isolates from lupin and chickpea, demonstrated about 50% foam stability for both proteins. Similar results were obtained by Pozani et al. [40]. The foaming properties of lupin proteins were also greater than those reported previously for soybean proteins (150% foaming capacity and 30% foam stability after 120 min), while being comparable to those of sunflower protein isolates (230% foaming capacity and 50% foam stability after 120 min) measured by the same procedures [30].

#### 3.3.3. Emulsifying

Regarding the emulsion properties (Figure 4c), the emulsion capacity was comparable (42–43%) among the tested lupin protein isolates, except for proteins extracted at pH 2 and purified via ultrafiltration (LPI-pH2/UF), which exhibited obviously lower abilities to form emulsion (32.6 ± 1.2%) compared to other isolates. Concerning the stability of emulsion against thermal coalescence, it was high and identical for all isolates (100.0 ± 0%). The correlation between emulsifying properties and isolation method has previously been studied by other authors. In line with our results, Chew and co-workers [14] have found a similar emulsion capacity for lupin proteins extracted in alkaline medium and purified by isoelectric precipitation or ultrafiltration. Likewise, the study by Makri et al. [41] revealed almost identical mean drop-size diameters for emulsions of lupin proteins at pH 7 prepared with these two purification methods. As in the present work, the stability of emulsion was high and similar among all lupin proteins.

### 3.4. Structural Analysis

**Figure 5:**
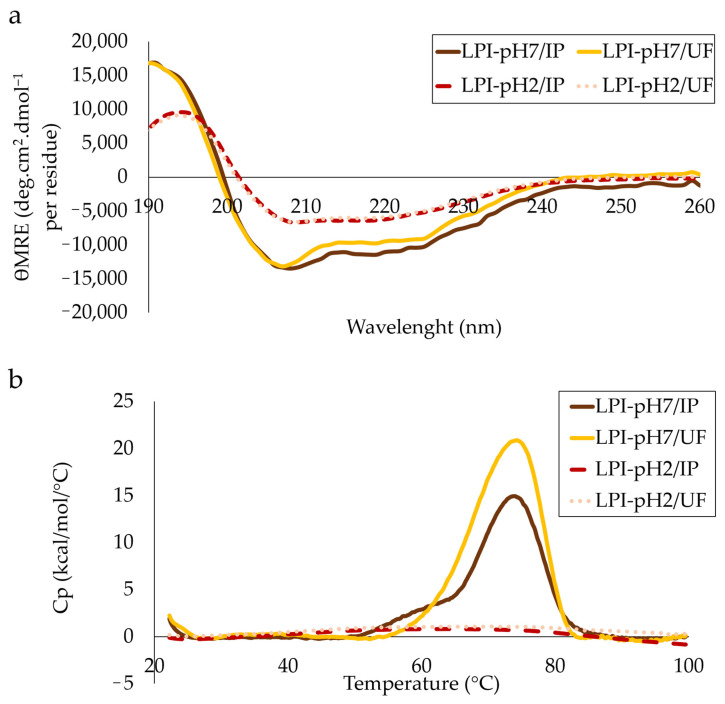
Far-UV CD spectra (190–260 nm) of lupin protein isolates at pH 7 (**a**). DSC scan of lupin protein isolates at pH 7, ranging from 20 to 100 °C (**b**).

The secondary structures of proteins from lupin isolates was analysed by circular dichroism. As shown in Figure 5a, far-UV CD spectra of isolates extracted at pH 7 (LPI-pH7/IP and LPI-pH7/UF) exhibited a maximum ellipticity near 190 nm and minimal ellipticity at 210–220 nm. These are characteristic spectra of globular protein with a high level of α-helix conformation. However, the CD spectra of LPI-pH2/IP and LPI-pH2/UF demonstrated an altered shape, with a lower contribution from the α-helix in the secondary structure. In general, the data for the composition of the secondary structures of lupin proteins are so far poorly reported in the literature. In line with our results, Lilley [42] has also shown that native δ-conglutin is primarily composed of the α-helix. However, this protein represents only about 10–12% of total lupin globulins. Further work by Alonso-Miravalles et al. [43] has examined the structural properties of lentil proteins prepared by isoelectric precipitation and ultrafiltration process. This study also revealed a well-defined α-helix secondary structure regardless of the purification method.

Regarding thermal properties of isolates (Figure 5b), LPI-pH7/IP and LPI-pH7/UF analysed at pH 7 showed single endothermic peaks of Tm at 74.0 and 74.4 °C, respectively. The enthalpies of the denaturation transition (ΔHcal) were 194.75 kcal.mol^−1^ for LPI-pH7/IP and 265.56 kcal.mol^−1^ for LPI-pH7/UF. The obtained results share a global conclusion with data from the literature. Indeed, Fontanari et al. [20], studying the thermal properties of white lupin protein isolates extracted under different extraction conditions and with isoelectric precipitation, found a denaturation temperature ranging from 63 to 74 °C. Similarly, Czubinski [44] determined a Tm of ϒ-conglutin at 71.1 °C as analysed in an aqueous solution at pH 7.5. Slightly different results were presented by Sirtori et al. [45], who observed two denaturation temperatures for lupin proteins at 71.5 and 90.9 °C, which were assigned to the thermal transition of vicilin-like 7S β-conglutin and legumin-like 11S α-conglutin, respectively. This was supported by the results from the work of Sousa et al. [46] and Muranyi et al. [11,21]. However, unlike the present study, these studies refer to other lupin species (*L. angustifolius* and *L. luteus*) that may differ in protein composition [47,48]. In contrast, the DSC thermograms of proteins extracted at pH 2 (LPI-pH2/IP and LPI-pH2/UF) were completely disordered without visible well-formed temperature peaks. Apparently, as was shown by the CD analysis, the extraction of lupin proteins at strong acidic pH causes protein denaturation and also results in poor structural resistance against thermal treatment.

**Figure 6:**
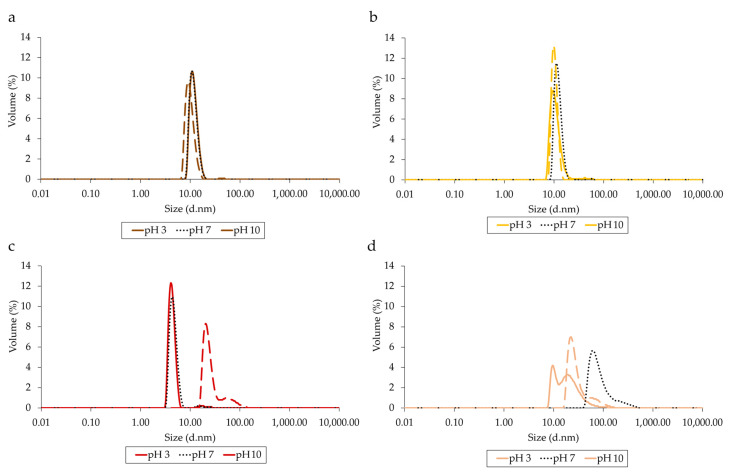
The volume–size distribution of lupin protein isolates at pH 2 (dashed line), 7 (solid line) and 10 (dotted line): LPI-pH7/IP (**a**), LPI-pH7/UF (**b**), LPI-pH2/IP (**c**) and LPI-pH2/UF (**d**).

The particle size distribution of lupin protein isolates as a function of pH (2, 7, 10) was evaluated by DLS analysis. As depicted in Figure 6, single peaks ranging from 5.5 to 10 d·nm for LPI-pH7/IP and LPI-pH7/UF were observed, which corresponded to the DLS distribution of plant proteins found previously by other authors [28,31]. Thus, from these data it can be concluded that lupin proteins extracted at pH 7 have a generally homogeneous particle size distribution in all studied pH, whatever purification method is applied. On the other hand, LPI-pH2/IP and LPI-pH2/UF were much more polydisperse, showing an additional shoulder greater in size than 100 d·nm. The large protein aggregations were predominantly formed at pH 7. Thus, these results provide further evidence confirming the denaturation of lupin proteins during the acidic extraction process.

Altogether, the results highlighted a loss of the native molecular state of lupin proteins exposed to the acidic medium used during the extraction process. As shown in many previous studies, plant globulins have generally lower stability across extremely low pH levels compared to more resistant albumins [28,49,50]. Consequently, the unfolding of the protein native structure and the exposure of hydrophobic amino acid residues may lead to a loss of some protein functionalities, such as solubility and properties on the interface. As demonstrated above, lupin proteins extracted at pH 2 have far lower solubility, in particularly close to neutrality, than proteins obtained at pH 7. This is probably due to the partial denaturation of these proteins. However, the observed modifications were apparently not significant enough to affect the foaming and emulsifying properties.

## 4. Conclusions

Extraction pH was found to be the most important parameter affecting the lupin protein yield. Interestingly, lupin proteins were highly extractable in acidic pH. Indeed, the extraction yield under these conditions was comparable to the conventional method of alkaline extraction (37% vs. 40–45%, respectively). The extracted proteins also showed similar protein profiles, the majority consisting of globulin fractions. In addition, the temperature had no significant impact on the extractability of lupin proteins, whereas lower extraction yields at pH 2 were found after NaCl addition (37% at 0 mol·L^−1^ NaCl vs. 15% at 0.25 and 0.5 mol·L^−1^ NaCl).

The isoelectric precipitation and ultrafiltration were then evaluated for purification of lupin proteins extracted at pH 2 and pH 7. As a result, four isolate-grade protein powders (91–100% on a dry matter basis) were produced with almost equal process yields ranging from 28.1% to 31.3%. All isolates had the same polypeptide profiles. However, further structural characterization revealed the partial denaturation of lupin proteins extracted under acidic medium, losses in the secondary structure, low thermal stability and the formation of aggregates. Similarly, proteins extracted at pH 2 had low solubility at pH 6 and 7 (10 and 50%). Other functional properties such as foaming and emulsifying were comparable overall across almost all isolates.

Therefore, the quality of produced lupin isolates seems to be mainly dependant on extraction pH, whereas the purification method does not appear to have a noteworthy effect on protein yield and properties. Lupin proteins were extracted at acidic pH with exceptionally high yields. However, despite the same composition, consisting of globulins, these proteins had altered structure and functionality. Thus, these results highlight the singular behaviours of lupin proteins during the extraction process. This phenomenon is particularly interesting with regard to the selective extraction of albumins in the strong acidic media of other conventional plant-based sources. Further studies should examine the precise impact of pH and NaCl concentration on protein structural properties in order to explain the mechanisms involved. It would also be revealing to investigate thoroughly the structural and functional properties of specific fractions of lupin proteins. This knowledge could be helpful for the future valorisation of lupin meal in human nutrition

## Figures and Tables

**Table 1:** Retention rate of lupin proteins and flux as a function of membrane cut-off during ultrafiltration.

Membrane Cut-Off (kDa)	Retention Rate of Proteins	Flux (mL/min/cm^2^)
10	1.00	0.09
30	0.99	0.09
100	0.99	0.09
300	0.97	0.11

**Table 2:** Process yields in the production of lupin protein isolates.

Process Yields	Lupin Protein Isolate			
	LPI-pH7/IP	LPI-pH7/UF	LPI-pH2/IP	LPI-pH2/UF
Extraction yield (%)	40.4	40.4	37.1	37.1
Purification yield (%)	66.5	72.5	75.8	76.1
Protein recovery (%)	30.2	32.2	28.1	31.3

**Table 3:** Colour of lupin protein isolates in solution expressed in the CIE L*a*b* scale.

Lupin Protein Isolate	Parameters of CIE L*a*b* Scale		
	L*	a*	b*
LP-pH7/IP	100.8	1.4	9.6
LP-pH7/UF	101.8	3.1	5.2
LP-pH2/IP	98.0	-0.2	8.1
LP-pH2/UF	101.0	2.8	2.9

## Data Availability

The data presented in this study are available on request from the corresponding author. The data are not publicly available due to the confidentiality of the project.
